# Molecular Mechanism of Tumor Cell Immune Escape Mediated by CD24/Siglec-10

**DOI:** 10.3389/fimmu.2020.01324

**Published:** 2020-07-17

**Authors:** Shan-Shan Yin, Feng-Hou Gao

**Affiliations:** Department of Oncology, Shanghai Ninth People's Hospital, Shanghai Jiao Tong University School of Medicine, Shanghai, China

**Keywords:** CD24, Siglec-10, tumor immune escape, immune surveillance, immunotherapy

## Abstract

Tumor immune escape is an important part of tumorigenesis and development. Tumor cells can develop a variety of immunosuppressive mechanisms to combat tumor immunity. Exploring tumor cells that escape immune surveillance through the molecular mechanism of related immunosuppression in-depth is helpful to develop the treatment strategies of targeted tumor immune escape. The latest studies show that CD24 on the surface of tumor cells interacts with Siglec-10 on the surface of immune cells to promote the immune escape of tumor cells. It is necessary to comment on the molecular mechanism of inhibiting the activation of immune cells through the interaction between CD24 on tumor cells and Siglec-10 on immune cells, and a treatment strategy of tumors through targeting CD24 on the surface of tumor cells or Siglec-10 on immune cells.

## Introduction

Normally, the existence of inhibitory receptors or immune checkpoints avoids the injuries caused by excessive immune response, as tumor cells can up-regulate the corresponding immune checkpoints and their ligands and inhibit the activity of immune cells or induce the apoptosis of immune cells, so as to escape the surveillance of the immune system (1). Tumor immune escape is one of the basic characteristics of tumor occurrence and development (2). The treatment of innate immune checkpoints related to tumor immune escape has achieved remarkable success in recent years. Therefore, the identification of innate immune checkpoints is very important in developing cancer treatment. In this regard, programmed cell death ligand 1 (PD-L1), cytotoxic T lymphocyte-associated protein 4 (CTLA-4), CD47, and some other innate immune checkpoints have been found (3–5). And the latest research has indicated that CD24 may be the dominant immune checkpoint in tumors. Its interaction with sialic-acid-binding Ig-like lectin 10 (Siglec-10) can promote tumor immune escape and is expected to become a new target for tumor therapy (6).

The CD24 gene is located on chromosome 6q21 and it encodes a glycosylated protein with 16 potential O-glycosylation and N-glycosylation sites (7). In general, CD24 is expressed on the surface of developing T and most B lymphocytes (8, 9). It can determine the ability of proliferation and survival of early T cells (10). Glycosylphosphatidylinositol (GPI) is required to bond with CD24 because the latter does not contain a cytosolic domain. That is why CD24 is also known as a heat stable antigen (7, 11, 12). The CD24 on immune cells adheres to the lipid raft as a cell adhesion molecule, so that it can participate in the transduction of signals such as tyrosine kinase, G protein, etc. (13, 14). CD24 is highly expressed in various tumor cells and is related to the occurrence and development, invasion, and migration of tumor cells (15–25). For example, CD24 is seen as a strong and independent molecular marker for the prognosis of ovarian cancer; it is also related to the growth and metastasis of breast cancer and may be related to the occurrence and development of pancreatic cancer (17, 26, 27). The combination of CD24-expressing tumor cells and P-selectin on platelets can promote the excretion of tumor cells from the bloodstream and thus promote their metastasis (28). Another tumor-related mechanism of CD24 is the connection between CD24 and signal factors in the lipid rafts microdomains, such as Src kinase. The Src kinase activated by CD24 may be involved in other mechanisms that cause tumorigenesis (25). For example, CD24 regulates the invasion of tumor cells by suppressing tissue factor pathway inhibitor-2 (TFPI-2) through a Src-dependent manner (29).

The sialic acid-binding immunoglobulin (Ig)-like lectins (Siglecs) are an immunoglobulin-like type I transmembrane protein with different numbers of Ig-like domains (C2 setting domain) and IgV-like domains that recognize the N-terminal of ligands (30). Siglecs have immune receptor tyrosine inhibitory motifs (ITIM) or ITIM-like motifs in cells, and many of them are related to protein tyrosines that contain an SH2 domain, like phosphatase 1 (SHP-1), and SHP-2 containing SH2 domain (31). Siglecs can recognize the sialic acid-containing structure and combine with the sialic acid attached to the glycoconjugates on the cell surface (31). In this siglecs family, ligand recognition results in an induction of accessibility of the cytosolic ITIM tyrosine and the ITIM-like tyrosine to Src family kinases (32). These kinases phosphorylate ITIM tyrosine in the cytoplasm, thereby recruiting tyrosine phosphatases such as SHP-1 or SHP-2, which can attenuate signal transduction (33, 34). Although SHP-1 and SHP-2 both belong to protein tyrosine phosphatases which contain the SH2 domain, they are usually regarded as negative regulators and positive regulators, respectively (35, 36). Siglecs can be divided into two groups according to their structure (30). The first group includes Siglec-1 (Sialoadhesin / CD169), Siglec-2 (CD22), Siglec-4 (Myelin-associated glycoprotein / MAG), and Siglec-15, which are structurally conserved in many species, such as mouse and human (30). The second group contains the CD33-related Siglecs, which are different in mouse and human. CD33-related Siglecs in humans are Siglec-3 (CD33),-5,-6,-7,-8,-9,-10,-11,-12,-14, and−16 while in the mouse Siglec-3(CD33), Siglec-E,-F,-G, and-H belongs to this group (30). Siglec-10 has five extracellular Ig-like domains, a transmembrane region, and a cytoplasmic tail containing two ITIM signaling motifs (37). The IgV structural domain of Siglec-10 contains a key arginine residue, which is related to the recognition of sialic acid (30). Siglec-10 is a kind of inhibitory receptor, which expresses widely in immune cells, such as B cells, monocytes, dendritic cells, a small number of NK cells, and a small subset of activated T cells which inhibit the function of immune cells (38–40).

**Figure 1:**
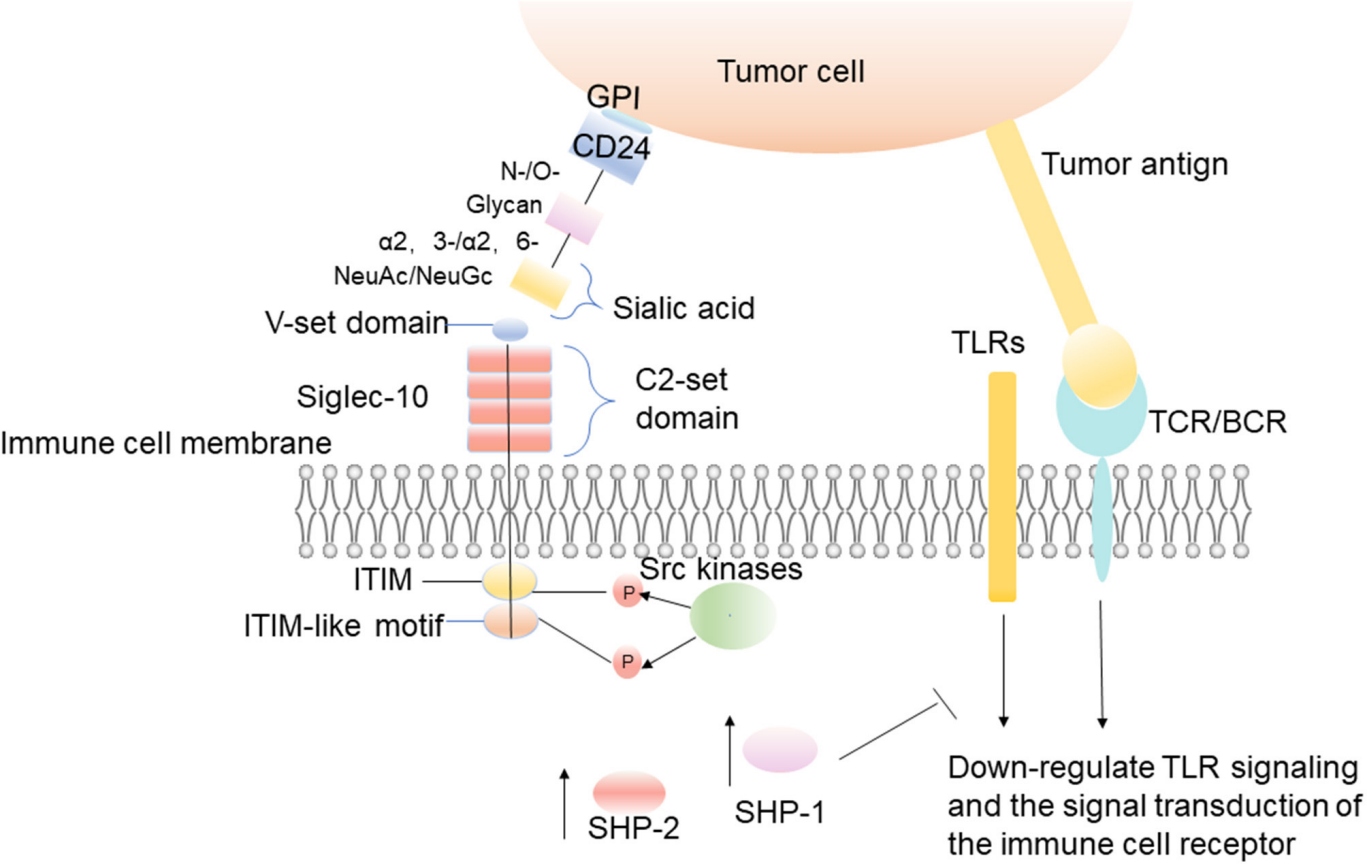
The interaction between CD24 on tumor cells and Siglec-10 on immune cells. The interaction between CD24 on tumor cells and Siglec-10 on immune cells results in inhibitory signal cascades. The IgV domain of Siglec-10 binds to the sialic acid located on the terminal region of CD24, which leads to the induction of Src family kinases by intracellular ITIM or ITIM-like motifs (31). These kinases phosphorylate ITIM tyrosine in the cytoplasm, and then recruit the tyrosine phosphatases such as SHP-1 and SHP-2 to reduce the signal transduction (31).

Siglec-10 binds firmly to CD24 in a sialylation-dependent manner, and CD24 is the main ligand of Siglec-10 (41, 42). When CD24 on tumor cells combines with Siglec-10 on immune cells, it causes the signal cascade of immune cell inhibition, which is mediated by SHP-1/SHP-2 (31). These phosphatases are associated with ITIM, which is in the cytoplasmic tail of Siglec-10. The ITIM region is phosphorylated, thus blocking Toll-like receptor (TLR)-mediated inflammation and activating a series of intracellular signal pathways to achieve effective immunosuppression and promoting tumor immune escape (Figure 1) (31, 43, 44). Existing studies show that the damage of danger-associated molecular pattern (DAMP)-associated inflammatory responses, which perform as innate immune pattern recognition receptors, can be reduced by the interaction of CD24-Siglec-10 (42). The interaction between CD24 and Siglecs is considered to be the complex of placental immunosuppressive response, and a great number of placental cells and molecular markers have been evaluated for their role in tumor immune escape (41).

### Recognition of CD24 by Siglec-10

Sialic acid is a family of nine-carbon sugars, which usually forms the terminal sugar structures of the glycoproteins and glycolipids on the cell surface (45). They connect carboxy on C-1, and connect the glycan via N- and O- on C-2 (46). Sialic acids can be linked to glycans by α 2-3-, α 2-6-, α 2-8-, and α 2- 9-linkage (46). Different sialic acid derivatives are formed according to the differences among modifications at C-5, which can be divided into four types: N-acetylneuraminic acid (Neu5Ac), N-glycolylneuraminic acid (Neu5Gc), deaminylneuraminic acid, and neuraminic acid (Neu) (47). The diversity within the sialic acid family itself and the linked- glycan, and the variable linkability of sialic acids, enables cells to synthesize and express a great variety of sialoglycans at the cell membrane (48, 49). The differences in the structure of each sialoglycan distinguish them from other sialoglycans. CD24 is a severely sialylated glycoprotein that can interact with Siglec-10 to escape immune recognition (45). It is always heavily decorated with N- and O-linked glycans (50, 51). Sialic acid is connected to glycans through α 2-3- and α 2-6-linkage (Figure 1). In the mouse brain, the glycans of CD24 are mainly complex type N-glycans and highly diverse patterns of O-glycans, including mucin-type and carrying O-mannosyl glycans (52, 53). It was noticed that structural features of sialic acids are important for Siglec binding (54). The sialic acid backbone can be chemically modified at various positions (54). Its chemical modifications of the sialic acid backbone can dramatically increase the binding affinity to a Siglec (55, 56). The carboxylic acid is crucial for Siglec binding and hence is left unmodified, but all other positions, ranging from the aglycone (C-2) to the rest of the backbone (C-3 to C-9), can potentially be modified to improve Siglec binding (54). The sialic acid in mouse brain CD24 is mainly NeuAc, and small amounts of NeuGc can be detected at the non-reducing end of mucin CD24 much-type O-glycans (Figure 1) (57). The type of linkage and type of underlying sugar also affects the recognition of sialic acids (54). Although all Siglecs can recognize sialoglycans, the binding preferences of these receptors vary considerably (54). Siglec-10 recognizes the sialic acid ligands carrying α 2-3- or 2-6-linkage (Figure 1). Siglec-10 can be attracted by the unique structure of the entire molecule of CD24 when it is binding to receptors.

## Molecular Mechanism of CD24 Expression in Tumor Cells

### CD24 Expression in Tumor Cells Induced by HIF1 α

The tumor cells undergo an exuberant process of metabolism and their oxygen consumption is high. Meanwhile, due to the shortage of oxygen supply, the oxygen content of the tumor microenvironment is low. In this condition, the tumor cells are in a state of relative hypoxia. Hypoxia-inducible factors (HIFs) are the most important proteins for cell-induced expression in hypoxic environments (58). Tumor cells use it to induce the expression of target genes to make tumor cells adapt to the hypoxic environment (59). The main reason for this is that under the action of normal oxygen, the proline hydroxylase hydroxylates those proline residues in the conserved region of HIF subunits, and VHL E3 ubiquitination ligase identifies and ubiquitinates the hydroxylated HIF so that the ubiquitinated HIFs can be degraded rapidly by the proteasome; however, under the condition of hypoxia, the prolinyl hydroxylase of HIF protein is inhibited, which stabilizes the protein level of HIF α (59, 60).

**Figure 2:**
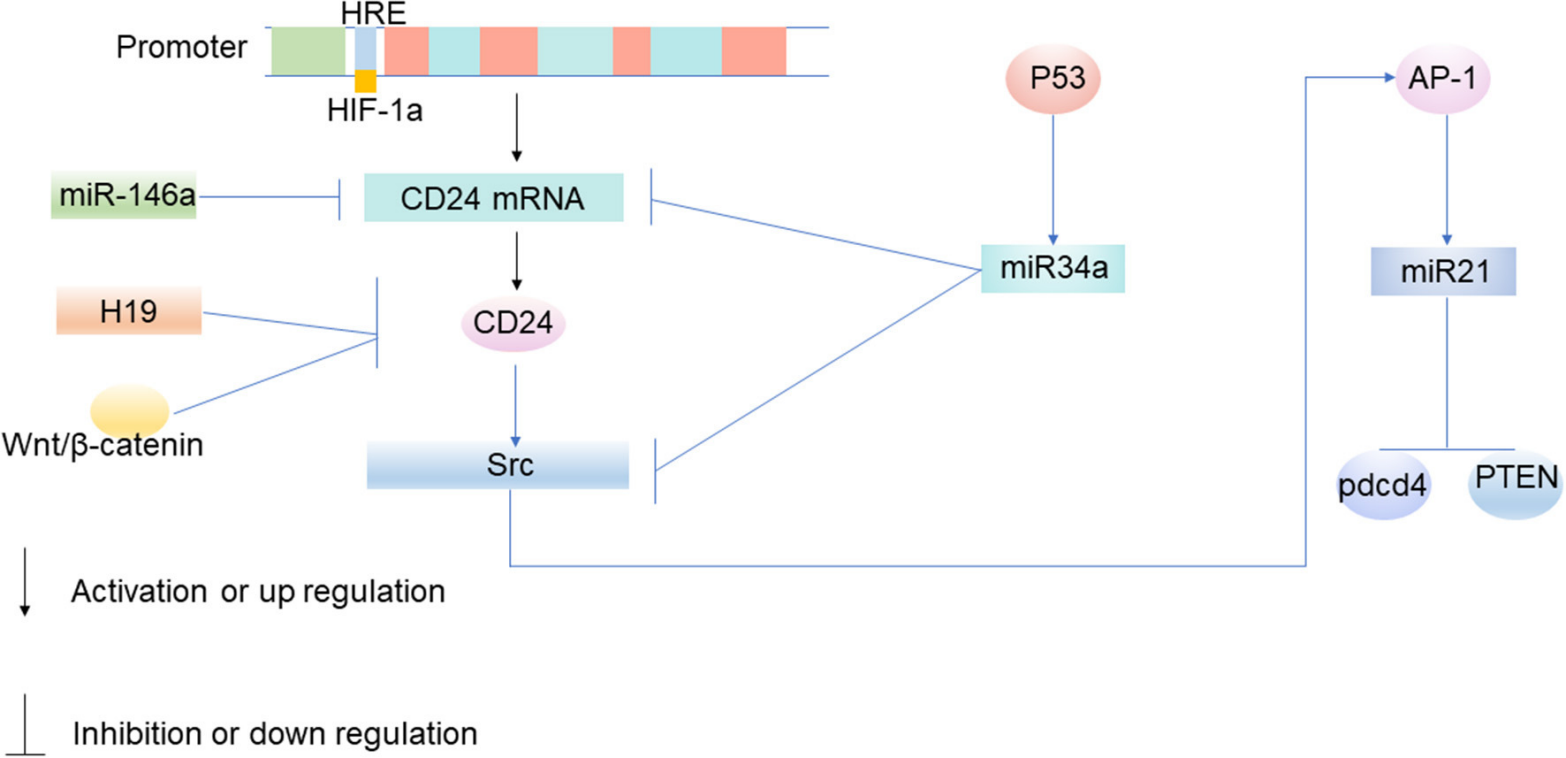
The molecular mechanism of CD24 expression in tumor cells. Under the condition of hypoxia, HIF-1α promotes the transcription of CD24 through a functional hypoxia-responsive element on the promoter of CD24 (61). MiRNA participates in tumorigenesis by participating in the upstream and downstream regulatory networks of CD24, and mainly inhibits the translation of mRNA. MiR-34a targets CD24 and Src at the post-transcriptional level, and inhibits the expression of CD24 and Src (62). When miR-34a is epigenetically silenced, the expression of CD24 up-regulates (62). The upregulation of CD24 expression can increase the expression of miR21 by activating Src, thus inhibiting the expression of Pdcd4 and PTEN, and participating in the occurrence and development of tumors (62–64).

Hypoxia-induced CD24 expression mainly occurs at the transcriptional level, especially when HIF acts as a transcription factor to induce CD24 expression (Figure 2). CD24 is the key transcriptional target of HIF-1α (61). HIF-1α promotes the transcription of CD24 through a functional hypoxia-responsive element in the CD24 promoter (61). In a previous study based on broad transcriptomic analysis of human umbilical cord vein endothelial cells exposed *in vitro* to hypoxia, Scheurer et al. reported that CD24 is one of the 65 genes that mRNA increases with hypoxia (65). In bladder cancer, prostate cancer, and gastric cancer, hypoxia significantly up-regulated the expression of CD24 mRNA and protein in cancer cells (61, 66).

### Non-coding RNA Induces CD24 Expression in Tumor Cells

Signals from non-coding RNAs (ncRNAs) can transfer between tumor cells and tumor microenvironments through extracellular vesicle (EVs), exosomes, and gap junctions (67, 68). NcRNAs have been demonstrated to play an important role in tumor growth, metabolism, and migration, as well as in regulating the expression of CD24. It has been found for the first time that the increased expression of long non-coding RNA (lncRNA) H19 leads to a reduction of cell-surface CD24, and that down-regulation of H19 helps to maintain the expression of CD24 on the cell surface, so H19 is thought to make a contribution to cell invasion by regulating CD24 expression, thereby regulating tumor immune escape (Figure 2) (69). MiRNA is also a kind of ncRNA. The protein-coding genes, such as the CD24 gene, are currently known to be regulated by miRNAs (62). Mature miRNAs regulate genes in two ways: one way is to bind to the target gene mRNA and promote its degradation, and the other is to inhibit the translation of mRNA (70). MiRNA participates in tumorigenesis by participating in the upstream and downstream regulatory networks of CD24, and mainly inhibits the translation of mRNA. MiR-34a targets CD24 and Src at the post-transcriptional level, and inhibits the expression of CD24 and Src (Figure 2) (62). When miR-34a is epigenetically silenced, the expression of CD24 up-regulates (62). CD24 is the direct target of miR-146a (71). MiR-146a binds to the 3'-untranslated region (UTR) of CD24 and suppresses its expression after transcription (Figure 2) (71).

### WNT/ β-catenin Induces CD24 Expression in Tumor Cells

The Wnt/ β-catenin signaling pathway is an evolutionarily-conserved regulatory pathway that governs numerous normal cellular and developmental processes such as cell fate determination, cell proliferation, and migration (72). However, aberrant Wnt signaling has also been identified as a key mechanism in cancer biology. It has been proven that Wnt/ β-catenin plays an important role in tumor growth and regulating the expression of CD24. Immunoprecipitation studies show that CD24 may activate β-catenin to interact with the Wnt pathway and induce β-catenin translocation into the nucleus (72). It has been shown in breast cancer that β-catenin can inhibit tumor immune escape by down-regulating the expression of CD24 (Figure 2) (73). And it has also been identified that CD24 is the transcriptional target of Wnt signaling in a non-transformed human mammary epithelial cell line MCF 10A (73).

## The Interaction Between Siglec-10 on Immune Cells and CD24 Induces Immune Escape of Tumor Cells

### T Cells

**Figure 3:**
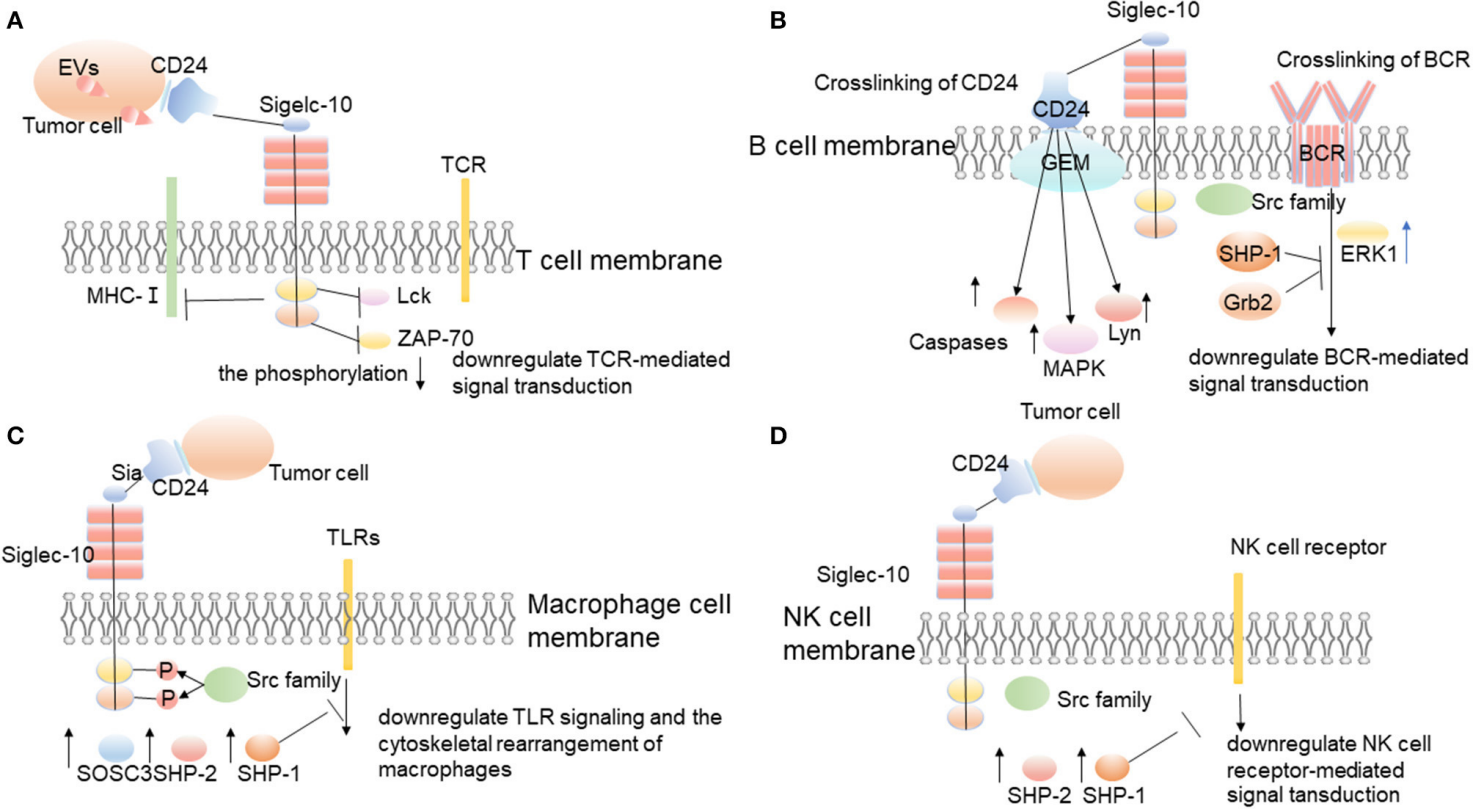
The interaction between CD24 and Siglec-10 on immune cells promotes tumor immune escape. CD24 expressed on the surface of tumor cells interacts with Siglec-10 expressed on immune cells, and then promotes tumor immune escape through corresponding molecular mechanisms. **(A)** Siglec-10 expressed on T cell surfaces inhibits the activation of T cells through inhibiting the formation of T cell major histocompatibility complex class I (MHC-I) peptide complex and the phosphorylation of T cell receptor-associated kinase, Lck, and ZAP-70 (39, 40, 75, 76). **(B)** Siglec-10 expressed on the surface of B cells inhibits BCR-mediated signal transduction. **(C)** When CD24 interacts with Siglec-10 on the surface of macrophages, it triggers the inhibitory signals cascade mediated by SHP-1 to inhibit the phagocytosis of macrophages and promote tumor immune escape (6, 31). **(D)** Siglec-10 expressed on the surface of NK cells inhibits NK cell receptor-mediated signal transduction.

Malignant cell-secreted Evs in the tumor microenvironment stimulate lymphocytes to suppress anti-tumor immunity and promote tumor progression. Importantly, malignant EVs impair T cells' function by upregulating the expression of Siglec-10 on T cells (74). Siglec-10 is an inhibitory receptor expressed on the surface of T cells (38, 39). It triggers immunosuppression by blocking the activation of TCR, which is realized by inhibiting the formation of T cell major histocompatibility complex class I (MHC-I) peptide complex and the phosphorylation of T cell receptor-associated kinases Lck and ZAP-70 (Figure 3) (40, 75, 76). Studies by Bandala-Sanchez et al. have also shown that Siglec-10 expressed on the T cells' surface inhibits the phosphorylation of T cell receptor-associated kinase ZAP-70 and the activation of T cells (39, 75). Siglec-10 can also inhibit T cells' activation by binding to corresponding ligands. For example, related studies by Sammar et al. have shown that CD52 (and possibly CD24, if properly glycosylated) can bind to Siglec-10 and block the activation of TCR by inhibiting T cell receptor (TCR)-related kinases (Figure 3) (41). CD24/Siglec-10 can inhibit the activation of T cells mediated by TCR and promote tumor immune escape.

### B Cells

Siglecs play an important immunomodulatory role in B cell activation and immunoglobulin production (77). For example, Siglec-2 strongly affects the signal transduction of the B cell receptor (BCR) and has become the prototype of the working principle of Siglec signal transduction (30, 78). B1 cells, the subtype of B cells, express the inhibitory receptor Siglec-10 in humans. However, it is still unclear how Siglec-10 regulates the activity of B cells (38). Siglec-10 not only expresses in human B cells, but also in mouse B cells, in which case it is referred to as Siglec-G. Siglec-G is an ortholog of human Siglec-10 (79). They have a high sequence identity, similar chromosomal location of their genes, and conserved structure of the proteins (79). Lineal homologs often have similar or even the same functions, which are regulated by a similar pathway, and play similar or even the same roles in different species (80–82). For example, both Siglec-10 in human cells and Siglec-G in mouse cells can combine with CD24 to inhibit host inflammation and immune response triggered by damage-related molecular models, and Siglec-10/G plays an important role in self-nonself discrimination of the immune system and may be involved in evasion of host immunity by RNA viruses (42, 45).

Existing studies have found that Siglec-G is an inhibitory receptor of B cells, which controls the proliferation and calcium signal transduction of B1 cells (83). Siglec-G is expressed in a B cell-restricted way, with large amounts present in B1 cells (83). When overexpressed, Siglec-G can inhibit B cell receptor-mediated calcium signaling (83). Siglec-G dampens the calcium signal transduction of B1 cells by recruiting the ITIM-binding protein SHP-1, growth factor receptor binding protein 2 (Grb2), and inhibits the activity of transcription factors NFATc1 and NF-kB (Figure 3) (45).

CD24 can also affect the function of B cells by affecting the signal transduction of BCR (Figure 3). CD24 induces human B cell apoptosis through glycolipid-enriched membrane (GEM) domains / raft-mediated signal transduction systems (12). The recruitment of a variety of signal transduction molecules in the GEM domain, including Src family PTKs, trimer G protein, Ras, and linker for activation of T cells, indicates their role as a signal transduction platform (84, 85). The association of CD24 and lyn protein tyrosine kinase in GEM enhances, and the activity of lyn also enhances, after CD24 cross-linking (12). In addition, after CD24 cross-linking, mitogen-activated protein kinases (MAPK) is activated, and CD24 mediates intracellular signal transduction that leads to B cell apoptosis (Figure 3) (12). The stromal-cell-derived factor-1 (SDF-1, also known as CXCL12) has a strong chemotactic effect on lymphocytes, and chemokine receptor CXCR4 is a specific receptor of CXCL12 (86). Using CD19-positive bone marrow B cells and CD24-/-Pre-B lymphocyte lines isolated from CD24 knockout mice proves that the expression of CD24 decreases CXCL12-mediated cell migration and signal transduction through CXCR4 (86). The study results suggest that CD24 mediates the apoptosis of human precursor B cells with the activation of multiple caspases in the pro-B and pre-B stages (87). The cross-linking of BCR precursors causes rapid and strong activation of extracellular signal-regulated kinase 1 (ERK1), while the cross-linking of CD24 induces continuous activation of p38MAPK after the activation of ERK1 (Figure 3) (87). Therefore, it can start the inhibitory signal, play its regulatory role, and promote tumor immune escape.

### The Interaction Between CD24 on the Surface of Tumor Cells and Siglec-10 on Macrophages Induces Tumor Immune Escape

The phagocytosis of macrophages to tumors is regulated by a host of signals, including pro-phagocytosis signals (“Eat me”) and anti-phagocytosis signals (“Don't eat me”) (88). Many phagocytic signals are expressed on the tumor surface, including tumor-associated antigen, endoplasmic reticulum chaperone, calreticulin, and glycoprotein signal lymphocyte activation molecule family member 7 (SLAMF-7; also known as CD319) (89–91). However, some anti-phagocytic signals also exist on the surface of tumor cells, including CD47, PD-L1, β 2-microglobulin (B2M), an unidentified ligand that binds to leukocyte immunoglobulin-like receptor-2 (LILRB2), and the recently discovered CD24 (6, 92–95). These “don't eat me” signals interact with the corresponding receptors on phagocytes surface, including signal regulatory protein α (SIRP α), programmed cell death 1 (PD-1), leukocyte immunoglobulin-like receptor 1 (LILRB1), Siglec-10, etc. The interaction between these receptors and ligands promotes the tumor to escape the phagocytosis of phagocytes. These anti-phagocytosis signals are all involved in macrophage signaling based on immunoreceptor-tyrosine-based inhibition motifs and essentially avoid the surveillance and clearance of macrophages (6). The researchers used gene knockout against CD24, Siglec-10, and monoclonal antibodies to block CD24 and Siglec-10, and then they found that macrophages increased their ability to engulf tumors and slow down the growth of macrophage-dependent tumors *in vivo* (6). And all the macrophages expressing Siglec-10 responded to the blocking of CD24, and the degree of these responses were related to Siglec-10. The loss of siglec-10 would decrease the blocking of CD24. It indicates that the specific blocking of CD24 occurs between CD24 and Siglec-10 (6). CD24 binds specifically to Siglec-10 but not to Siglec-3 and Siglec-5 (41). The interaction between CD24 and Siglec-10 triggers the inhibitory signal cascade (Figure 3) (45). After SRC family tyrosine kinases phosphorylate the cytoplasmic tyrosine-based signal transduction group, Siglec-10 recruits and activates the proteins containing the SH2 domain, especially SHP-1, SHP-2, or suppressor of cytokine signaling 3(SOCS3) (Figure 3) (31). As an important member of the tyrosine phosphatase family, SHP-1 can specifically bind to tyrosine phosphorylated in the intracellular ITIM domain and catalyze its dephosphorylation. It can also negatively regulate the intracellular signal transduction in which the growth factors, cytokines, hormones, extracellular matrix, and cell adhesion molecules are involved (96). Therefore, the interaction between CD24 and Siglec-10 inhibits the phagocytosis of macrophages, so that the tumor cannot be cleared by phagocytosis, which promotes the immune escape of tumors.

Healthy normal tissues and cells have the inherent ability to avoid the self-elimination of macrophages by expressing anti-phagocytosis molecules, but cancer cells rely even more on similar mechanisms to escape the eradication of immune-mediated (97–100). Therefore, the targeted therapy toward the macrophage phagocytosis checkpoints in tumors may provide a new avenue for the development of cancer immunotherapies to eliminate tumor immune escape (101).

### NK Cells

In liver tumor microenvironments, Siglec-10 mainly expresses on NK cells, while it expresses less on T cells and B cells (102). The percentage of Siglec-10+NK cells in tumor tissues is higher than that in surrounding non-tumor tissues (40). The high expression of Siglec-10 on NK cells can mediate the functional damage of NK cells in human hepatocellular carcinoma (HCC) (40). According to the results of survival analysis, the increased expression of Siglec-10 in HCC is negatively correlated with the prognosis of patients with HCC (40). The interaction between CD24 and Siglec-10 can repress the tissue damage-induced immune responses (42). And the interaction between CD24 expressed by hepatoma cells and Siglec-10 expressed by NK cells may be beneficial for tumors to escape the killing effect of NK cells and promote tumor immune escape (Figure 3) (40).

## The Strategy of Targeting CD24/SIGLEC-10 to Inhibit Tumor Immune Escape

### Antibodies of Targeting CD24

SWA11 monoclonal antibody has high affinity and specificity toward CD24-expressing cells (21). CD24 is internalized in cells after the binding of SWA11mAb, and the role of the SWA11 monoclonal antibody is mainly to reduce the proliferation of tumor cells (21, 103). SWA11mAb targeting CD24 effectively retarded the growth of lung and ovarian carcinoma xenografts (103). Dual treatment of pancreatic adenocarcinoma cells with anti-CD24 mAb and cetuximab enhanced phagocytosis relative to either treatment alone, demonstrating a potential synergy between anti-CD24 mAb and anti-solid-tumor mAbs. Besides, the addition of anti-CD24 antibody to the chemotherapy regimen may be beneficial to target chemotherapy-resistant tumor stem cells (103). However, potential off-target effects of anti-CD24 mAb treatment in humans include the depletion of B cells, owing to high CD24 expression by B cells (6). Meanwhile, the potential toxicity of targeted CD24 to cancer patients cannot be ruled out at this stage (6).

### Targeting CD24 and Siglec-10 Genes

**Figure 4:**
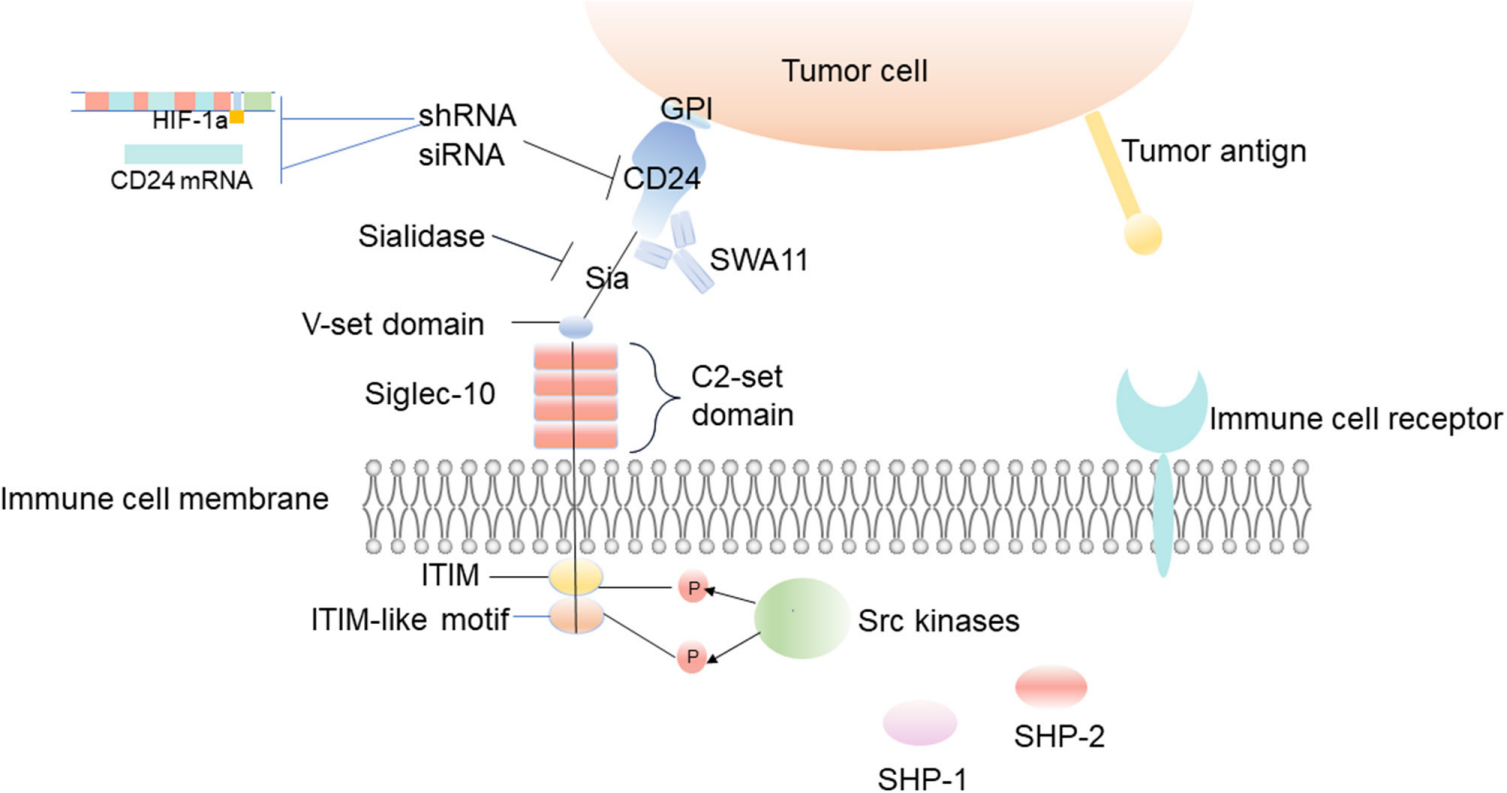
Inhibition of tumor immune escape by interfering with CD24/Siglec-10 signal transduction. The uses of CD24 monoclonal antibodies or sialidase is designed to interfere with the interaction between CD24 and Siglec-10, block the transduction of inhibitory signals, and inhibit the immune escape of the tumor. And the decrease of HIF-1α or CD24 expression is also a potential method for the treatment of inhibiting the immune escape of tumors.

Through gene ablation of CD24 and Siglec-10, the targeting of these cells has been proven to be an effective method to enhance the phagocytosis of macrophages (Figure 4) (6). Knockdown of CD24 expression by CD24-shRNA can significantly inhibit cell viability and induce the apoptosis of SKOV3 cells (104). Administration with CD24-shRNA *in vivo* suppressed tumor volume increase by microvessel density (MVD) decrease, cell proliferation inhibition, and apoptosis induction, suggesting that knockdown of CD24 may be a potential method for the treatment of human ovarian cancer (104). When CD24 targeted siRNA molecules are added to the growth medium of several epithelial cancer cell lines, such as breast cancer and prostate cancer, the transient low expression of CD24 leads to the decrease of cell growth, and the changes of actin cytoskeleton can be observed, which results in exercise damage (18).

### The Binding of Targeting CD24-Siglec-10

Siglec-10 is thought to have the ability to recognize sialic acid structures, and it binds firmly to CD24 in a sialic acid-dependent manner (43, 44). The latest research shows that loss of tumor sialic acid can block the effect of immune modulatory Siglecs on immune cells (105). It has been reported that the antibody-sialidase conjugates are used to edit the glycocalyx accurately, and the antibody guides sialidase to selectively remove sialic acid from tumor cells, which enhances the sensitivity of tumor cells to antibody-dependent cell-mediated cytotoxic (ADCC) and enables immune cells to kill desialylated cancer cells (106). For example, treating CD24 with sialidase abrogates the interaction with Siglec-10 and CD24 (Figure 4) (107). Barkal et al. also observed that the binding of Siglec-10 Fc (Fc, crystallizable fragments) to MCF-7 cells decreased significantly after desialylation on the cell surface (6). Blocking the interaction between CD24 and Siglec-10 with a monoclonal antibody can robustly augment the phagocytosis of human tumors expressing CD24 (6).

### Other

CD24 on the surface of tumor cells can also be regulated by other factors to promote tumor immune escape. HIF-1a induces the expression of CD24 at the transcriptional level to promote tumor immune escape, and the non-coding RNA, Wnt/ β-catenin, promotes or inhibits the expression of CD24 to promote or inhibit tumor immune escape (61). Although the effects of hypoxia on tumor growth and metastasis have been known for a long time, recent studies show that hypoxia can also promote tumor immune escape. Current studies suggest that HIF-1α can induce the expression of CD24 at the transcriptional level, which further points out the importance of hypoxia and the expression of CD24 for tumor immune escape (61). The decrease of HIF-1α or CD24 expression mediated by shRNA reduces the survival rate of cancer cells *in vivo* and *in vitro* at the growth level of primary and metastatic tumors (Figure 4) (61). Down-regulating HIF-1 can improve the sensitivity of chemotherapy and inhibit tumor formation. Therefore, inhibiting these CD24-related upstream molecules of regulatory signaling pathways can effectively prevent tumor invasion and immune escape, improve the tumor microenvironment, and may have a positive effect on tumor treatment.

## Author Contributions

F-HG handled the conceptualization and the writing and editing of the review. S-SY handled the writing of the original draft.

## Conflict of Interest

The authors declare that the research was conducted in the absence of any commercial or financial relationships that could be construed as a potential conflict of interest.
